# National Library of Medicine Disaster Information Management Research Center: Achieving the vision, 2010–2013

**DOI:** 10.3233/ISU-140731

**Published:** 2014

**Authors:** Cynthia B. Love, Stacey J. Arnesen, Steven J. Phillips, Robert E. Windom

**Affiliations:** a Disaster Information Management Research Center, Specialized Information Services Division, National Library of Medicine, National Institutes of Health, U.S. Department of Health and Human Services, Bethesda, MD, USA; b Division of Specialized Information Services, National Library of Medicine, Bethesda, MD, USA; c U.S. Department of Health and Human Services, Sarasota, FL, USA

**Keywords:** National Library of Medicine, Disaster Information Management Research Center, history, preparedness, response, recovery, medicine, disasters, public health emergencies, emergency management, libraries, librarians, informationists, Bethesda Hospitals’ Emergency Preparedness Partnership, hazards, databases, Internet, Web, research and development, WISER, REMM, CHEMM, Disaster Lit, Resource Guide for Disaster Medicine and Public Health, Resource Guide for Public Health Preparedness, National Network of Libraries of Medicine, NN/LM

## Abstract

From 2010 to 2013, the National Library of Medicine (NLM) Disaster Information Management Research Center (DIMRC) continued to build its programs and services on the foundation laid in its starting years, 2008–2010. Prior to 2008, NLM had a long history of providing health information, training, and tools in response to disasters. Aware of this legacy, the NLM long range plan (*Charting a Course for the 21st Century: NLM’s Long Range Plan 2006–2016*) called for creation of a center to show “a strong commitment to disaster remediation and to provide a platform for demonstrating how libraries and librarians can be part of the solution to this national problem”. NLM is continuing efforts to ensure that medical libraries have plans for the continuity of their operations, librarians are trained to understand their roles in preparedness and response, online disaster health information resources are available for many audiences and in multiple formats, and research is conducted on tools to enhance the exchange of critical information during and following disasters. This paper describes the 2010–2013 goals and activities of DIMRC and its future plans.

## 1. Introduction

This paper reviews the 2010–2013 progress of the Disaster Information Management Research Center (DIMRC) toward achieving the vision of its establishment at the National Library of Medicine (NLM). It builds on a 2013 paper [22] that describes the establishment and early years of DIMRC in 2008–2010 and reviews NLM disaster-related activities prior to 2008.

Based on the recommendations in *Charting a Course for the 21st Century: NLM’s Long Range Plan 2006–2016* [6], NLM has made significant progress in establishing the Disaster Information Management Research Center. In just a few years, DIMRC has developed, enhanced, and maintained several key resources and tools; created NLM’s first mobile applications; experimented with health information technology for hospital disaster response; and developed key collaborations with a number of agencies and organizations.

DIMRC activities and current initiatives include:

- Roles for Librarians, Information Specialists and Libraries
  - Training and supporting librarians to serve as Disaster Information Specialists in meeting their communities’ needs through continuing education, social media, and networking.
  - Funding disaster information outreach projects that require a partnership between a library and an institution with disaster responsibilities.
- Literature and Terminology: Supporting Disaster-Related Research
  - Collecting, organizing, and disseminating health information for all stages of preparedness and response to disasters.
- Tools and Resources for the Disaster Workforce
  - Developing innovative products and services to serve emergency responders.
  - Developing disaster medicine and public health online resources for health professionals.
  - Raising awareness and providing training in the use of NLM’s disaster health resources, and providing training on their use.
- Health Information Technology
  - Conducting disaster-related informatics research and development projects to support disaster health information management and healthcare facilities preparedness and response.
  - Ensuring that tools and resources are available in a variety of formats, using the latest technology commonly in use.
- Outreach and Partnerships
  - Collaborating with other federal agencies, state and local communities, public health officials, and emergency managers in efforts to prevent, respond to, and reduce the adverse health effects of disasters.
  - Partnering with other government agencies involved in disaster health to ensure information needs receive adequate attention in planning for disasters and are included in training for responders.
- Incident Response
  - Providing guides to disaster medicine and public health information resources for specific incidents, such as the Haiti earthquake and cholera outbreak; the Gulf of Mexico oil spill; the earthquake, tsunami, and radiation incident in Japan; and Hurricane Sandy.
  - Collaborating with NLM, its eight Regional Medical Libraries, and 6,000+ member-libraries of its National Network of Libraries of Medicine (NN/LM) [31] in planning and training for continuity of operations for library facilities, services and staff.

This paper explores each of these areas of interest and also includes guidance provided by an advisory group established by the NLM Board of Regents and by the DIMRC strategic plan (2012–2015).

## 2. National Library of Medicine disaster-related activities: History and background

NLM, one of the National Institutes of Health (NIH) and part of the U.S. Department of Health and Human Services (HHS), has played a pivotal role in translating biomedical research into practice since 1836. NLM, with more than 20 million books, journals, manuscripts, audiovisuals and other forms of medical information, is available to people worldwide via Web resources such as PubMed [42] and PubMed Central [43] references and full-text journal literature for professionals, and MedlinePlus [25] health information for the public. As a research institute, NLM also includes performing and funding biomedical informatics research as a major function.

NLM has long provided health information, training, and tools in response to disasters. The NLM commitment to providing disaster health information pre-dates the creation of the Disaster Information Management Research Center by several decades and is documented in a 2013 paper [22] on the Center’s “Establishment and Growth, 2008–2010”. Pre-2008 efforts included the development of WISER (Wireless Information System for Emergency Responders) [55], REMM (Radiation Emergency Medical Management) [45], and the Influenza Virus Resource [21]; assistance for Hurricane Katrina-affected medical libraries; disaster-related informatics research grants; and the formation of the Central American Network for Disaster and Health Information (CANDHI, now known as LANDHI or RELACIGER) [44]. NLM encourages greater recognition of information management and access to health information resources as key components of disaster medicine and public health.

As it has for more than 175 years, NLM collects and disseminates medical information through both its comprehensive online resources and its print, audiovisual, and historical collections. For example, on disaster-related topics:

- PubMed includes nearly 60,000 medical journal articles on disaster topics from 5,700 journals, including 40+ journals exclusively on disaster and emergency medicine.
- MedlinePlus, a Web site for the public, has over 40 subject pages on disaster and all-hazards topics in both English and Spanish.
- The NLM catalog [30] lists well over 1,000 publications on disaster topics related to medicine, including, for example, dozens on the 2011 Japan earthquake, tsunami, and nuclear incident.
- TOXNET [51] is a collection of databases on toxicology, hazardous chemicals, and environmental health that provides comprehensive information on thousands of chemicals, maps of facilities releasing or containing toxic chemicals, and more. It is an invaluable source for preventing and responding to hazardous materials incidents and is the backbone of many NLM tools for responders.

## 3. About the Disaster Information Management Research Center

As described in the earlier paper [22], the Disaster Information Management Research Center was created in response to recommendations of the NLM Board of Regents’ Long Range Plan (2006–2016) directing NLM to support national emergency preparedness and response efforts. Following a year and a half of planning, DIMRC opened in July 2008 with a staff of seven as part of the Specialized Information Services (SIS) Division [48] in Bethesda, Maryland. DIMRC coordinates disaster-related activities across NLM and works collaboratively with a number of other federal agencies on disaster medicine and public health information management activities (see Outreach and Partnerships section).

The DIMRC Web site [8] is the primary source for information about the center, disaster health information resources, NLM emergency preparedness and response tools, medical and scientific literature, partnerships, research activities, and Disaster Information Specialist Project libraries and librarians.

DIMRC works with the NLM Board of Regents, senior staff, and its many partners to determine goals, priorities, and users’ needs. Through DIMRC’s many activities, NLM demonstrates that libraries and librarians can be part of the solution to national disaster preparedness, response, and recovery efforts and makes a strong commitment to ensure continuous access to health information when disasters occur. Major activities include:

- DIMRC monitors and responds to current events, such as Hurricane Sandy, by selecting and disseminating timely, authoritative sources of needed information.
- To supplement the peer-reviewed journals in PubMed and the MedlinePlus materials for the general public, DIMRC maintains a database of grey (non-commercial) literature for medical and public health professionals.
- DIMRC and other branches in SIS provide hazmat/CBRN (chemical, biological, radiological, and nuclear) emergency response tools. The Radiation Emergency Medical Management (REMM) system gives health care providers guidance on the diagnosis and treatment of radiation injuries. The Wireless Information System for Emergency Responders (WISER) contains information on handling chemical, biological, and radiological incidents, including a decision support tool to aid in the identification of unknown chemicals. CHEMM (Chemical Hazards Emergency Medical Management) contains information on mass casualty chemical incidents for first responders, health care professionals, and planners.

As part of its 2012–2014 strategic planning, DIMRC identified its mission:

To develop and provide access to health information resources and technology for disaster prepared-ness, response and recovery.

and its vision:

Connecting people to quality disaster health information and fostering a culture of community resiliency.

The strategic plan outlined this vision of DIMRC’s ideal future:

- DIMRC serves as the expert and authoritative source for reliable disaster health information management.
- Health professionals, responders, and policymakers have the right information at the right time to prepare for, respond to, and recover from disasters.
- Librarians and other partners across the Nation are skilled in disaster information outreach to their communities.
- DIMRC is a diverse team of subject matter experts, information specialists, and computer scientists collaborating to collect and organize the best disaster health information around the world.
- DIMRC provides access to quality information using innovative tools and technology on multiple platforms.
- Information specialists are recognized as essential participants in disaster preparedness, response and recovery.
- DIMRC conducts and supports world-class research and develops innovative solutions.
- DIMRC has the resources to sustain long-term mission success.

Three Senate Reports (2008, 2010 and 2012), written in support of appropriations for NLM, have recognized DIMRC activities [41]. The most recent report stated:

“The Committee [on Appropriations] applauds NLM’s support of the Disaster Information Management Research Center, [DIMRC] which has made important contributions to the Nation’s disaster preparedness and response efforts through the rapid creation of information resources for specific events; development of innovative information tools to aid in disaster preparedness, response and recovery; the establishment of a disaster information specialty among librarians; and its participation in the Bethesda Hospital Emergency Preparedness Partnership. The Committee encourages NLM to continue to work through DIMRC with federal and non-federal partners, including the library community, to identify and implement best practices for maintaining access to health information before, during, and after disasters; develop innovative resources and tools to aid emergency responders and managers; collect, organize, and disseminate disaster health information; promote the development of disaster information specialists; engage the library community in disaster information management; conduct research into disaster health informatics; and develop and export community-based models of health resiliency during disasters”.

### 3.1. Guidance for DIMRC

DIMRC is guided by outside advisors and internal strategic plans. The NLM Board of Regents, the Library’s advisory board [29], established an ad hoc Working Group on the Disaster Information Management Research Center in 2010. The Group, chaired by Robert Windom, M.D., met once in 2011 and once in 2012. The all-day meetings had wide ranging discussions that included NLM staff presentations on past and current DIMRC initiatives. Group members, from multiple disciplines and all with disaster experience, are listed in Appendix A.

The Board of Regents posed the following questions in its charge to the Working Group:

1. What opportunities to advance information management in disaster medicine and public health play to NLM’s strengths and capabilities?
2. Are the current activities of DIMRC useful to the disaster medicine and public health community?
3. What future activities are recommended for NLM regarding disaster medicine and public health informatics research?
4. How can NLM strategically integrate with existing programs in the public and private sector regarding disaster preparedness, response, and recovery both domestically and internationally?

The Group’s guidance is highlighted in the sections below on “Future Considerations” and in a final report. In response to the Group’s guidance, DIMRC staff conducted planning sessions in late 2011 and 2013 to develop and update a strategic plan (described above) for 2012–2014.

## 4. Roles for librarians, information specialists and libraries

NLM continues to explore new roles for librarians in supporting the information needs of disaster medicine, public health preparedness, and emergency response. A new role, the Disaster Information Specialist, functions as an informationist or clinical librarian providing research and knowledge management in disaster medicine. NLM encourages librarians to be more proactive in working in this field and learning about the information tools, apps, and other resources needed by the emergency/disaster workforce. Multiple roles for librarians and informationists have been identified. For example, librarians can assist in many information management activities before, during, and after disasters, including assisting with data collection and analysis, database enhancement and maintenance, identifying and disseminating information, and using social media for rapid communication and situational awareness.

In a very important change for libraries, the Robert T. Stafford Disaster Relief and Emergency Assistance Act [20] in 2011 named libraries as providing essential community services and thus eligible for temporary relocation if their facilities are affected by a disaster. This modification highlights the key role of libraries as contributors to community resiliency. DIMRC reaches out to libraries and library associations to promote an understanding of this change in the Stafford Act and its implications for public libraries.

The NLM Disaster Information Specialist Program supports the role of specialists by offering training programs [52] and tools to enhance their skills and keep up to date. The training program began with a Disaster Information Outreach symposium [10] held at NLM in March 2011. A Medical Library Association (MLA) Disaster Information Specialization certificate of accomplishment is now offered by MLA for those who complete a series of classes and other activities outlined by DIMRC. Following the model of core competencies in disaster medicine developed for many health professions, DIMRC developed core competencies for Disaster Information Specialists as documented in the Disaster Information Model Curriculum [9]. The core competencies were based in part on work [54] by the National Center for Disaster Medicine and Public Health [26] and the American Medical Association (AMA) Center for Public Health Preparedness and Disaster Response. The Disaster Information Specialist curriculum covers key concepts from the disaster medicine and public health field for librarians, as well as health information literacy for the disaster workforce. Six courses (each three to four hours) were developed and offered both in-person and online in 2011–2012 and are archived for on-demand use. The courses were developed and taught by librarians and other subject matter experts.

At the 2011 NLM Disaster Information Outreach Symposium, several researchers presented their findings [11] on librarians’ activities in response to disasters, interest in continuing education on disaster information topics, results from library student coursework on disaster information, health information needs of the disaster workforce, and hospital staff needs for H1N1 information. Evaluation methods included surveys and interviews of librarians, library school students, U.S. Public Health Service Commissioned Corps members, emergency planners in Pennsylvania, and hospital staff in Canada.

Following the first year (2012) of continuing education provided by the Disaster Information Specialist Program, students were surveyed on their participation in the program and plans for implementing their training [38]. In addition, evaluations of instructors and course content were collected following every class for immediate feedback. The same evaluation form was also collected after the 2011 Symposium and after every presentation by staff on DIMRC resources [50].

NLM provides ongoing support to Disaster Information Specialists through an information outreach listserv and monthly webinar calls for librarians and information specialists that aim to build a community of practice and partnerships. The DISASTR-OUTREACH-LIB listserv [14] has over 1,250 subscribers. DIMRC has hosted over 60 webinar calls with an average attendance of 75 people [12]. A bibliography of news, interviews, and journal articles [28] features librarians’ disaster-related efforts. DIMRC has sponsored and benefitted from several NLM Associate Fellow Projects [18].

The Disaster Information Specialist concept is equally directed at librarians and those in the disaster workforce who play similar information roles in their agencies. The vast majority of staff and volunteers playing information roles related to disasters are not librarians or informationists. They are responders who have a particular interest in or responsibility for organizing information and implementing the latest information technology. Responders need to be fully aware of the best disaster health information resources and tools. DIMRC structures its resources, outreach, and training materials to reach this crucial audience as well. It relies on interested librarians and NN/LM staff around the country to provide training on NLM disaster resources to these local responders. Subscriptions to the DISASTR-OUTREACH-LIB listserv have shifted dramatically from mostly librarians (65 percent at first) to mostly responders (now 65 percent). DISASTR-OUTREACH-LIB content (news, announcements, discussions) is widely reposted to channels reaching responders.

DIMRC also sponsors the Disaster Health Information Outreach and Collaboration Projects [35] to explore potential roles for Disaster Information Specialists. Seventeen projects have been funded in three years, with each project requiring the active participation of a library. See the section below on Outreach and Partnerships for more about these projects.

Social media has grown more prominent in disaster management both for communications between the public, news media, and responder agencies, and for monitoring situational awareness of an unfolding event. DIMRC maintains a Twitter presence, @NLM_DIMRC [1], exchanging tweets daily about its resources and current events with its 2,500 followers, many of whom are disaster-related organizations or part of the disaster workforce. DIMRC has sought to inform both librarians and the disaster workforce about the skills librarians can contribute to social media and disasters, including skills useful for data collection and synthesis, validation of information sources, and tracking of requests for assistance. On its monthly webinar calls, DIMRC has hosted speakers on the Digital Operations Center [2] at the American Red Cross national headquarters, VOST (Virtual Operations Support Team), and the HHS Office of the Assistant Secretary for Preparedness and Response (ASPR) fusion center’s use of social media to raise awareness of new and interesting ways to explore social media data. DIMRC staff have presented at international and national events on the importance of social media in disasters, both for information sharing and for analysis to improve situational awareness and response.

### 4.1. Future considerations for librarians and disaster information specialists

Based on the recommendations of the DIMRC Working Group and strategic plan, DIMRC will continue to work in the following areas to promote roles for librarians, information specialists and libraries:

- *Continue to promote contributions of the Disaster Information Specialist for disaster preparedness, response and recovery*.

DIMRC will continue to be proactive in demonstrating the value of Disaster Information Specialists, and extend training beyond medical librarians to other librarians and to the disaster workforce (perhaps through the FEMA Emergency Management Institute Independent Study Program [19] or training resources like Pacific EMPRINTS [39]). DIMRC will consider adding a practicum component to the training program. Disaster Information Specialists should become increasingly proactive in assisting disaster/emergency personnel through education/training and knowledge management. Suggested routes for promotion include attending conferences and publishing articles and editorials about the Disaster Information Specialist concept in both library journals and journals that reach an emergency/disaster workforce audience. Areas to explore are marketing the Specialist concept to public health departments and policymakers, and suggesting that librarians support Emergency Operations Centers; assisting public health departments and emergency shelters with information for responders, shelter operators, and the public; collaborating with local health and hospital hotlines during a crisis; and working with poison control centers and local disaster voluntary organizations.

- *Support the exploration, adoption and use of information-sharing tools*.

DIMRC, with support from the Working Group, is investigating how social media can be used in disaster management. DIMRC will work with other agencies to investigate how to evaluate the use of social media for disaster preparedness and response in order to understand user information-seeking behaviors. Librarians should use and promote relevant technologies to increase community resiliency.

## 5. Literature and terminology: Supporting disaster-related research

The NLM mission is to collect, organize, and disseminate the world’s biomedical literature.

DIMRC works with other NLM divisions to ensure that PubMed adequately covers the disaster medicine and public health journals. PubMed includes nearly 60,000 references to disaster medicine and public health articles, and indexes 40+ disaster/emergency medicine journals. To enhance retrieval and improve standardization, NLM indexing terms related to disasters were reviewed, and substantial additions were made to MeSH (Medical Subject Headings).

Much of the disaster health information for professionals is not published in journals or other commercial publications [53] and can be described as grey literature. Based on his experience in editing a recent book, a Working Group member provided an illustration of the need for identifying grey literature by noting that 85 percent of the book’s references were to grey literature [4]. DIMRC has a key focus on managing this grey literature that is diverse, not well organized or easy to find, and perishable. It includes reports from state and local health departments, publications by non-governmental organizations, guidelines, toolkits, evaluations, field assessments, after-action reports, exercises and drills, lessons learned, training classes, multimedia training materials, and Web sites. The target audience for such materials is the disaster medicine and public health workforce, including responders, healthcare providers, public health officials, policymakers, and trained volunteers. NLM ensures that information on disasters for the general public is also available via MedlinePlus with links to many consumer-friendly sources such as Ready.gov and Flu.gov.

DIMRC has revamped and enhanced the former “Resource Guide for Public Health Preparedness” and renamed it “Disaster Lit: the Resource Guide for Disaster Medicine and Public Health” or simply “Disaster Lit”. The scope of and approved publication sources for the database have been completely reviewed and updated. Materials are selected from about 800 sources, 10% of which are the most important and prolific producers of disaster health grey literature. Significant improvements have been made in the search engine, the display and filtering of results, indexing, and topic coverage. New additions to the database are widely announced. Disaster Lit remains unique in its extensive coverage of disaster-related medicine and public health in comparison to other databases on disaster topics.

Disaster Lit is gaining recognition as the primary source of grey literature in the field. Other agencies have asked DIMRC to include content from small, niche databases that can no longer be supported and to expand the scope and coverage of Disaster Lit to host information they want to make widely available. DIMRC is working with the National Institute of Environmental Health Sciences (NIEHS) to include records describing post-disaster data collection instruments selected for the NIH Disaster Research Response Project [32]. Records from the PedPrepared (Pediatric Disaster Resource Clearing-house) database of the Emergency Medical Services for Children National Resource Center [15] have been transferred to Disaster Lit for permanent archiving.

Access to information during and following disasters is critical, and NLM has worked with a number of publishers to create the Emergency Access Initiative (EAI) to provide free access to over 65 books and databases and over 200 key medical journals in times of disasters. The Emergency Access Initiative has been opened for several events: the earthquake and subsequent cholera outbreak in Haiti (2010); the floods in Pakistan (2011); the earthquake, tsunami, and nuclear incident in Fukushima, Japan (2011); and the Typhoon Haiyan flooding in the Philippines (2013). Designated NLM staff and publishers’ representatives determine when to activate EAI.

### 5.1. Future considerations for literature and terminology

Based on the recommendations of the DIMRC Working Group and strategic plan, DIMRC will continue to work in the following areas to ensure access to the disaster medicine and public health literature:

- *Continue to refine the scope and coverage of the peer reviewed journal literature, grey literature, and books; identify needed areas of coverage beyond the core health sciences*.
  - Encourage the publication of evaluations and outcomes from non-governmental organizations (NGOs). NGOs may be especially reluctant to publish less-than-favorable evaluations and outcomes of their services as they are dependent on donations.
  - Determine the level at which DIMRC can include information in Disaster Lit as literature is published at local, state, national, and international levels for both government agencies and NGOs.
  - Continue to refine selection criteria.
  - Identify literature on health aspects of disasters that is in publications from other disciplines, outside medicine or public health. DIMRC will investigate how to access this information as well, perhaps using a source aggregator tool.
  - Investigate the issues involved in archiving the full text of electronic documents that might otherwise disappear (perishable information).
  - Investigate whether NLM could play a role in synthesizing literature reviews or collaborate with Evidence Aid, [17] which already does this following the Cochrane Collaboration model.
  - Investigate the feasibility of creating special collections of online materials for selected disaster events.
- *Investigate the development of an organized and simplified terminology modeled on MeSH and/or the Unified Medical Language System (UMLS)*.

Some Working Group members were concerned about the inconsistent and poorly-defined terminology used in the published research literature on disaster medicine, especially its use by those involved only sporadically in disaster response. Although this is an issue best addressed by journal editors and researchers, NLM supported the World Association for Disaster and Emergency Medicine (WADEM) initial investigation of ways to develop a common disaster medicine research vocabulary. NLM also identified over 60 existing English-language glossaries, dictionaries, and controlled vocabularies in this field [7].

## 6. Tools and resources for the disaster workforce

DIMRC uses its information management and information technology expertise to create tools and organize health information for the disaster workforce. NLM packages health information in a variety of formats based on the needs and roles of the disaster workforce. Information is compiled through several methods and then delivered on a range of devices, including posting online and downloading to desktops/laptops and mobile devices. Content is derived from TOXNET, PubMed, MedlinePlus and other authoritative data. The goal is to provide vital information to protect responders as well as treat patients, and to educate responders on health effects and medical management that are unfamiliar to many healthcare providers.

DIMRC has developed several tools for preparing for and responding to hazardous materials (hazmat) and chemical, biological, radiological and nuclear (CBRN) events. WISER, launched in 2004, contains information on the handling and treatment of chemical, biological, and radiological incidents, including a decision support tool to aid in the identification of unknown chemicals, information from NLM’s Hazardous Substances Data Bank and the Department of Transportation 2012 *Emergency Response Guidebook*. WISER is now available as an app on Apple iOS, Android, and Blackberry tablets and mobile devices, in addition to the Web and as downloaded files to laptop/desktop computers. There have been nearly half a million downloads to the various devices (iOS, Android, and Blackberry) in addition to Web access.

Chemical Hazards Emergency Medical Management (CHEMM) debuted on the Web in the summer of 2011 and contains information for first responders, health care professionals, and emergency planners on mass casualty chemical incidents. At the request of users, CHEMM was incorporated into the WISER iOS and Android apps. CHEMM was built by NLM in conjunction with ASPR and subject matter experts from the Department of Homeland Security and the National Institute of Child Health and Human Development, emergency responders, and other healthcare providers.

A third tool, Radiation Emergency Medical Management (REMM), provides health care providers with guidance on the diagnosis and treatment of radiation injuries. REMM was developed in conjunction with ASPR, the Centers for Disease Control and Prevention (CDC), National Cancer Institute, and NLM, and became publicly available in 2007. Following the March 2011 Fukushima earthquake, REMM was used by HHS representatives assisting the Japanese government in the management of the radiation release from a damaged nuclear power plant, and portions of REMM were translated into Japanese. Like WISER and CHEMM, REMM is available on the Web, can be downloaded to desktop/laptop computers, and is a mobile app for iOS, Android and Blackberry devices.

For the past two years, DIMRC has been working with ASPR to develop an interactive All-Hazards Plan on the Web for use in the HHS Secretary’s [Emergency] Operations Center during response to disasters and public health emergencies. NLM used its information management and technology skills to repackage a static document into a searchable, interactive tool that allows for quick identification of activities based on the timeline of the incident, responsible agency, and tasks required. The interactive All-Hazards Plan includes pre-established options and recommended actions to support coordination of the Federal Emergency Support Function #8 – Public Health and Medical Services [16] response.

Mobile apps are proving to be invaluable tools for providing crucial information to responders during a disaster. As mobile apps evolved, DIMRC moved quickly to create versions of its responder tools for smartphones and tablets, developing the first mobile apps offered by NLM. DIMRC is also collaborating with the U.S. Food and Drug Administration (FDA) to develop an app for healthcare providers to report adverse events related to use of medical countermeasures during a public health emergency. During the H1N1 pandemic, the antiviral drug Peramivir was given Emergency Use Authorization by FDA [40]. However, there was no quick and easy mechanism to collect data on possible adverse events from the use of this drug. An app may make it easier and more likely that providers will report adverse events to FDA during future public health emergencies.

Software for archiving Web pages and social media content may prove useful for developing “lessons learned” after a disaster. Following major incidents, collections of materials about the event are often started for historical and educational purposes. Universities and libraries in the disaster-affected region are the most likely institutions to take on this archival task, sometimes starting long after an event. With the enormous proliferation of transient materials on the Internet related to each disaster, it becomes a huge challenge to capture and archive news stories, situation reports, videos, photos, and self-published materials (blogs, tweets, Facebook postings) that give a comprehensive picture of an event as it unfolds. The NLM History of Medicine Division (HMD) and DIMRC have experimented with archiving materials about the H1N1 pandemic, at the request of ASPR. As Internet-archiving software and search engines become more sophisticated, there will undoubtedly be opportunities to revisit these methodologies for use in capturing the stories of future disasters in permanent repositories.

All of the tools and content for the disaster workforce can be found on the DIMRC Web site, as well as many resources of particular interest to librarians. DIMRC offers a wide perspective on the topic of disaster health information and includes pages on disaster glossaries, medical subject headings, bibliographies, disaster and emergency medicine journals, and other topics on accessing and organizing disaster health literature. Training courses and materials are online, along with core competencies from many health professions. DIMRC staff have compiled dozens of topic guides both on specific disaster incidents and on health issues related to types of disasters (pandemic influenza, hurricanes) or common to many types of disasters (coping with stress, planning for mass gatherings, environmental hazards during recovery). During a specific disaster, the topic page for that event is usually the most frequently visited DIMRC Web page.

### 6.1. Future considerations for tools and resources for the disaster workforce

Based on the recommendations of the DIMRC Working Group and strategic plan, DIMRC will continue to work in the following areas to ensure the availability of authoritative, timely resources for the disaster workforce:

- *Develop information search and retrieval systems and related tools using current technologies and platforms to assist in disaster preparedness, response and recovery*.

The Working Group was especially interested in identifying gaps in available resources and healthcare provider knowledge of disaster medicine, and tailoring tools to address those needs. It recommended considering the use of federated search tools to identify content from multiple sources, ways to display retrieval, and methods to get feedback from users on DIMRC resources.

- *Support for the exploration, adoption and use of information-sharing tools*.

The Working Group suggested creating syntheses of available information through the use of new concepts for authoring that allow for multiple contributors, continuous updating, and a level of peer review. Social media, particularly the Wikipedia and crowd-sourcing models, may be promising approaches. DIMRC will consider investigating these tools to enhance resources and encourage interaction and collaboration among the disaster workforce.

## 7. Health information technology

Since 2008, NLM has been a leader in initiating and supporting research and development in biomedical informatics, communications systems, and resources that create and provide access to information relevant to disaster preparedness, response, and recovery. These projects have been conducted by DIMRC, other NLM divisions, and other researchers. The Bethesda Hospitals’ Emergency Prepared-ness Partnership (BHEPP) received funds specifically for research and development. These were used to launch several new projects to assist the participating hospitals in preparing for and responding to major incidents. NLM developed prototype redundant communications systems and tools to aid hospitals in patient management, family reunification, and disaster response training. Products and prototypes have been tested at annual mass casualty drills held by the three Bethesda hospitals and in other settings [36]. The BHEPP projects and earlier NLM grants are highlighted in the 2008–2010 paper [22]. Despite the discontinuation of funds for BHEPP, several prototypes continue to be further enhanced and tested with BHEPP and new partners. The challenge for DIMRC will be transitioning prototypes into operational tools.

NLM is an early adopter of new technologies and uses its information management and technology skills to investigate new ways to collect, organize, and deliver information. Resources once available only on the Web are now available with responsive design or as versions specifically developed for use across multiple mobile platforms, as discussed in the previous section. For example, a nearly real-time system for family reunification was developed as part of the BHEPP initiative. ReUnite, a mobile app, allows for the collection of information on “found” people, such as those in shelters or hospitals following a disaster. The information is then uploaded to the Lost Person Finder Website which can share data with other family reunification systems. ReUnite was developed following the 2010 earthquake in Haiti. In the aftermath, it became clear that there was a need to easily collect information for local use and wide distribution. NLM is working with other federal agencies and NGOs on how to incorporate the Lost Person Finder with other tools, and has tested the tool at several disaster drills.

DIMRC has also been experimenting with virtual reality software by developing a virtual reality training tool that uses avatars (3-D characters) and interactive role playing to allow BHEPP hospital personnel to practice their roles within the Hospital Incident Command System (HICS). Developed in the virtual world Second Life, the tool was used by hospital staff for two disaster management exercises based on a hospital evacuation scenario inspired by events during Hurricane Sandy. Plans are now underway to develop a single-player system for staff to learn to navigate in the virtual world and conduct a small exercise to test navigation skills by performing HICS tasks.

### 7.1. Future considerations for health information technology

Based on the recommendations of the DIMRC Working Group and strategic plan, DIMRC will continue to work in the following area to ensure the development and effective use of health information technology to enhance disaster preparedness, response, and recovery:

- *Experiment using the latest generation health information technology tools, including mobile devices, social media, telemedicine and virtual reality*.

NLM will consider expanding efforts to determine how the latest information technology tools may assist other hospitals or organizations involved in disaster preparedness, response, and recovery. NLM will also ensure that existing tools are adapted to current technologies used by the disaster community.

## 8. Outreach and partnerships

DIMRC promotes awareness and use of NLM resources by all relevant communities, including emergency/disaster managers and planners, healthcare workers, emergency responders, disaster volunteers (American Red Cross, Community Emergency Response Teams, Medical Reserve Corps and others), and librarians/information specialists.

Shortly after DIMRC was created, it joined the Bethesda Hospitals’ Emergency Preparedness Partnership [3]. This Congressionally-funded partnership was a unique experiment in joint preparedness and response activities among private, military, and federal hospitals, plus NLM, in the Maryland suburbs of Washington, DC. NLM was included in the partnership to address information, communication, and technology needs (as described above).

DIMRC has made substantial progress in building on existing partnerships and identifying new ones in the public and private sectors, both domestically and internationally. Outside the United States, DIMRC has worked most closely with the Pan American Health Organization (PAHO) and the Regional Disaster Information Center for Latin America and the Caribbean (CRID). Since 2001, NLM and CRID have developed and funded a collaborative network that has grown from six library centers in three countries in Central America to 15 library centers in 11 Central and South American countries. The centers expand access to Spanish-language health and disaster management information and are partners in supporting the growth of a disaster risk reduction culture in their regions. They serve government entities, NGOs, community organizations, educational institutions, and the general public in Latin America.

Within the United States, DIMRC works with a number of federal agencies to develop, enhance, and expand access to information resources and tools. DIMRC enhances and supports their roles via information and communication resources, tools, and research. The federal agencies include:

- Department of Health and Human Services
  - Office of the Assistant Secretary for Preparedness and Response.
  - Centers for Disease Control and Prevention.
  - Food and Drug Administration.
  - National Cancer Institute (NCI).
  - National Institute of Child Health and Human Development (NICHD).
  - National Institute of Environmental Health Sciences.
  - National Institutes of Health Clinical Center.
- Department of Homeland Security.
- Department of Transportation (DOT).
- Walter Reed National Military Medical Center.

NLM/DIMRC also serves on disaster-related and public health emergency committees, including:

- Institute of Medicine Forum on Medical and Public Health Preparedness for Catastrophic Events.
- ASPR Council on Emergency Care.
- Trans-National Institutes of Health Committee on Biodefense Research.
- Federal Education and Training Interagency Group.
- Intra-NIH Disaster Interest Group (I-DIG).

To reach potential partners at the state and local level, NLM funds Disaster Health Information Outreach and Collaboration Projects. Seven projects were funded each year in 2011 and 2012; three projects were funded in 2013. This program brings together disaster health organizations (hospitals, academic health centers, non-governmental organizations, emergency response agencies, local health departments and others) and libraries to develop unique ways to use the information tools and resources of libraries and the skills of librarians to assist all types of disaster/emergency workers with their information needs. Awardees have included academic health centers, a community hospital and hospital alliance, public health departments, public libraries, and a national professional association. The funds have been used for developing resources, providing training, supplementing collections, and developing relationships to enhance knowledge of and access to disaster health information in all phases of disasters. The projects are intended to benefit equally the libraries and their partners who are required to be organizations with disaster responsibilities. Through these local projects, the project leaders are introducing DIMRC resources and ideas as part of their reporting to state and regional government officials and at meetings and conferences. Disaster-related projects proposed by libraries are also among the many projects selected for funding by the NN/LM Regional Medical Libraries.

DIMRC staff and NN/LM Regional Medical Library staff frequently give presentations on DIMRC resources for a range of audiences, both in-person and online with an emphasis on “disaster health literacy 101”. Staff also provide training classes, posters, and selected papers at national disaster medicine and public health–related conferences, as well as major library conferences.

### 8.1. Future considerations for outreach and partnerships

Based on the recommendations of the DIMRC Working Group and strategic plan, DIMRC will continue to work in the following areas to ensure continued progress in outreach efforts and partnerships:

- *Continue to develop partnerships with disaster/emergency agencies and organizations*.

DIMRC will continue developing partnerships and look to new agencies and organizations. In addition to the agencies listed above, the Working Group suggested working with FEMA, the Public Health Service, University of Pittsburgh Supercourse [49], private and public sector agencies responsible for water quality and other environmental issues, the military’s National Center for Telehealth and Technology [27], and professional associations and public health organizations such as the Association of State and Territorial Health Officials (ASTHO) and the National Association of City and County Health Officials (NACCHO).

- *Engage in outreach activities with relevant communities*.

NLM will continue to explore ways to teach the medical and public health workforce about disaster-related information resources, as recommended by the Working Group. In addition, DIMRC will explore outreach to the library community beyond the medical librarians via the American Library Association and the Special Libraries Association.

- *Continue efforts to evolve a discipline of disaster medicine and public health preparedness, and identify core competencies*.

The Working Group recommended that NLM continue to work with the National Center for Disaster Medicine and Public Health at the Uniformed Services University of the Health Sciences, and the AMA Center for Public Health Preparedness and Disaster Response (whose efforts are now being continued by the Society for Disaster Medicine and Public Health [47]) on the development of core competencies and curricula for the wide variety of health professionals involved regularly or ad hoc in disasters. DIMRC especially advocates for the inclusion of competencies that address disaster health information literacy [54] for medical and public health professionals and also compiles the many core competencies that have been published for health disciplines [13]. NLM, as it does for all medical disciplines, identifies and disseminates outcomes-based and evidence-based research in disaster medicine.

## 9. Incident response

Although DIMRC has no officially assigned disaster response duties within the National Response Framework, the Center is responsive to the many major regional, national, and international incidents that arise. Following an incident, DIMRC provides timely, authoritative health information for use in affected communities; encourages local librarian participation in response efforts; and exploits teachable moments to further prepare librarians in unaffected regions. DIMRC especially focuses on unfamiliar topics and rare occurrences such as massive oil spills, radiation incidents, or incidents that cause illness or injuries not encountered in routine medical practice. The NN/LM Regional Medical Libraries are invaluable partners in disseminating information about disaster health issues when a disaster strikes in their regions. They also reach out to librarians affected by a disaster and to libraries experiencing major service disruptions.

The Center responded to the 2010 Gulf of Mexico oil spill; the 2010 Haiti earthquake and cholera outbreak; the 2011 Japan earthquake, tsunami, and radiation incident; interest in novel viruses (H7N9, Middle East Respiratory Syndrome Coronavirus [MERS-CoV]); drought conditions in North America; major U.S. wildfires and tornadoes; school and workplace mass shootings; Hurricane Sandy (2012); and, in 2013, Typhoon Haiyan in the Philippines, the West, TX fertilizer facility explosion, and the Boston Marathon bombing. Depending on the incident, DIMRC response may include wide dissemination of health information to responders and librarians through Twitter, the DISASTR-OUTREACH-LIB list-serv, webinars, and Web guides or topic pages pointing to incident-specific online resources. A hands-on approach was taken by a senior computer scientist from DIMRC asked by PAHO to help create a local emergency operations center in Port-au-Prince and organize information about the activities of the nearly 100 agencies providing health care in Haiti during the cholera epidemic. DIMRC coordinates with other federal agencies to provide government-wide consistent, authoritative health information relevant to each incident. Announcements of activation of the Emergency Access Initiative (for free access to books, journals, and databases) are widely distributed. DIMRC staff monitor public news sources and HHS Secretary’s Operations Center reports and response activities to maintain awareness of public health issues stemming from an incident.

In the aftermath of specific disasters, NLM is the only library that is proactive in national distribution of relevant health information through pre-established channels. Federal agencies that also provide health information in response to an incident (including CDC, FDA, ASPR, Environmental Protection Agency, and Department of Energy) almost always concentrate on their own resources and do not aggregate the information from all appropriate sources for the primary benefit of health workers. DIMRC is unique in aggregating health information from a wide range of sources and in providing ready access (preformatted searches) to relevant materials from PubMed, Disaster Lit, TOXNET, and other NLM databases at the time of a disaster.

## 10. Hurricane Sandy case study

As an example of incident response by DIMRC and NN/LM Regional Medical Libraries, the following efforts were undertaken immediately after and in the months following Hurricane (Superstorm) Sandy on October 29, 2012:

- The Web-based All-Hazards Plan developed by NLM was successfully used for operational responses in the HHS Secretary’s Operations Center during Superstorm Sandy.
- The DISASTR-OUTREACH-LIB listserv carried 37 messages about Hurricane Sandy posted by DIMRC and others, starting the day before landfall.
- DIMRC created two new topic pages, one immediately after the event (November 5), http://sis.nlm.nih.gov/dimrc/hurricanesandy.html, and one for the recovery phase, http://disasterinfo.nlm.nih.gov/dimrc/hurricanesandyrecovery.html
- Related topic pages were updated: Floods, Hurricanes, Disaster Recovery and Environmental Health, and Coping with Disasters.
- DIMRC staff followed news about Hurricane Sandy to identify and share information about emerging health issues and status of healthcare facilities.
- NLM resources were widely promoted, including through *NLM in Focus* and *NLM News & Events*, 139 tweets on the DIMRC Twitter account, the Middle Atlantic Regional Medical Library blog, the NN/LM Bringing Health Information to the Community blog, and the NN/LM Emergency Pre-paredness Toolkit.
- DIMRC consulted with the affected NN/LM Regional Medical Libraries (Middle Atlantic, Southeastern Atlantic and New England) about how to respond, and contacted affected libraries/librarians.
- The Middle Atlantic Regional Medical Library produced a webinar, Sharing Experiences and Lessons Learned from Hurricane Sandy, on March 28, 2013 [46].
- The monthly DIMRC webinar calls following the hurricane included discussion by librarians in affected regions. The speakers from ASPR on the December 2012 call used Hurricane Sandy as a case study for social media response.
- Thirty news stories about response to Hurricane Sandy were collected for the *Librarians and Libraries Respond to Disasters* bibliography [28].
- Ports in a Storm, a conference in Eatontown, NJ on April 8, 2013, was coordinated by the NN/LM Middle Atlantic Region (which also provided $9,000), the NN/LM Emergency Preparedness and Response Initiative, LibraryLinkNJ, the New Jersey Library Cooperative, and the New Jersey Library Association. DIMRC and NN/LM staff spoke at this conference for New Jersey librarians.
- Ports in a Storm: Your Library as a Disaster Recovery Center. A New, Community-focused Approach to Library Disaster Planning, a preconference held June 28, 2013 in conjunction with the American Library Association annual meeting, was organized by the NN/LM Emergency Prepared-ness and Response Initiative (coordinator Dan Wilson) and the New Jersey Library Association. This national event was modeled on the success of the April 2013 New Jersey conference.
- NLM Director’s Podcast featured Hospital Readiness for Hurricane Sandy on January 7, 2013 [34].
- The Middle Atlantic Regional Medical Library funded four Hurricane Sandy-related projects for 2012–2013 [23] and five for 2013–2014 [24].

Of course, as in any incident, it is the local response that has an impact on those directly affected. The local libraries and librarians in the affected areas of New Jersey and New York played many creative and generous roles in their local communities. Some of these libraries were damaged or destroyed by the hurricane or surrounded by community devastation. DIMRC and the NN/LM played a very modest supporting role compared to those on the frontlines. The local school, public, and hospital libraries’ efforts are well documented and were greatly appreciated. Examples of their contributions can be found in the *Librarians and Libraries Respond to Disasters* bibliography as well as in photos, [33] media reports, video [37], libraries’ blogs and newsletters, and efforts to collect the stories of those impacted by the storm [5].

## 11. Conclusions

DIMRC’s mission is *To develop and provide access to health information resources and technology for disaster preparedness, response, and recovery by connecting people to quality disaster health information and fostering a culture of community resiliency*.

Based on the recommendations in *Charting a Course for the 21st Century: NLM’s Long Range Plan 2006–2016*, NLM has made significant progress in establishing the Disaster Information Management Research Center. In just a few years, DIMRC has developed, enhanced, and maintained several key resources and tools; created NLM’s first mobile applications; experimented with health information technology for hospital disaster response; and developed key collaborations with a number of agencies and organizations.

DIMRC aims to assist other government agencies in providing information in a coordinated, just-what-I-need, just-in-time fashion, and does not seek to duplicate efforts assigned to other agencies. The focus of these agencies is on the creation of the material, whether it is writing and updating guidelines in response to an emerging disease, such as H1N1 (CDC); announcing the authorization of a drug for emergency use in a public health emergency (FDA); or providing critical medical treatment information in response to a chemical spill, radiation leak or improvised nuclear device. NLM has the expertise to provide assistance in organizing this information and making all related information easier to find; and to develop innovative tools for locating and presenting the information.

NLM has an excellent reputation in the medical community for developing high-quality, authoritative health information resources. With the formation of DIMRC, NLM has continued to demonstrate its skill in providing a wealth of resources to serve multiple needs and users. DIMRC is a new organization that has good results in reaching and informing a wide variety of emergency/disaster preparedness personnel including firefighters and EMTs, emergency managers, and public health specialists. Outreach to these disparate groups will require constant attention by DIMRC staff and others involved in health information outreach such as staff and members of the National Network of Libraries of Medicine. DIMRC also will need to expand and explore its use of new technologies for communication, promotion, and community engagement, such as social media. DIMRC’s Twitter account has 2,500 followers and has been at the forefront of NLM’s efforts to engage with our followers and related organizations and not use social media solely as a self-promotion tool.

Over the past several years, DIMRC has collaborated with a number of agencies and organizations, including ASPR, NIH (NCI, NICHD, NIEHS, Clinical Center), FDA and DOT. DIMRC worked with these agencies to develop databases, Web resources, and mobile apps. These collaborations show good cooperation among government agencies to decrease duplication and enhance easy access to critical information. However, DIMRC’s expertise in the development of health IT tools and complex information management is not widely known in the disaster management community. DIMRC will need to engage with other developers and emergency/disaster personnel (DHS Science and Technology Directorate and FEMA; state and local public health and emergency management agencies) to demonstrate existing tools and ways in which NLM staff (librarians, computer scientists, and subject matter experts) can assist in the research and development of new tools to aid in disaster preparedness and response.

Community resiliency is the goal of Presidential Policy Directive-8. Libraries and librarians are at the heart of communities and are seen as trusted sources of information, safe environments for learning, and “essential community services”. NLM is the leader in defining roles for librarians to assist in all phases of disasters, and training librarians in the available tools and resources to assist disaster personnel in their communities, as well as in ensuring continuity of operations at libraries themselves. To gain and retain a “seat at the table” (meaning working closely with emergency management staff and decision-makers), DIMRC will need to expand the Disaster Information Specialist concept and promote the varied and technically sophisticated tools and resources it has developed, as well as demonstrate its willingness to listen, learn, assist, and participate in activities related to enhancing community resiliency.

## Appendix. NLM Board of Regents Working Group (2011, 2012) on the Disaster Information Management Research Center

**Table:**

CHAIR	EXECUTIVE SECRETARY
**The Honorable Robert E. Windom, MD** (2011–2012) Consultant Former Assistant Secretary for Health, HHS 1986–1989 windom@comcast.net	**Steven J. Phillips, MD** (2011–2012) Director, Specialized Information Services National Library of Medicine phillips@nlm.nih.gov
**MEMBERS**	
**Marvin Birnbaum, MD, PhD** (2011–2012) Editor-in-Chief, Prehospital and Disaster Medicine Past-President, World Association of Disaster and Emergency Medicine Professor Emeritus, University of Wisconsin Mlb@medicine.wisc.edu	**James J. James, MD, DrPH, MHA** (2011–2012) Director, Center for Public Health Preparedness and Disaster Response Editor-in-Chief, Disaster Medicine and Public Health Preparedness American Medical Association James.James@ama-assn.org
**Frederick “Skip” M. Burkle, MD, MPH. DTM, FAAP, FACEP** (2012) Senior Fellow and Scientist Harvard Humanitarian Initiative Harvard School of Public Health Senior Associate Faculty and Research Scientist Center for Refugee and Disaster Response Johns Hopkins University Skipmd77@aol.com	**Catherine N. Norton, MLS** (2011–2012) Director, Marine Biological Laboratory Library Woods Hole, MA cnorton@mbl.edu
**The Honorable S. Ward Casscells, III, MD** (2011) Vice President for External Affairs and Public Policy at the University of Texas Health Science Center, Houston, TX Former Assistant Secretary of Defense (Health Affairs), 2007–2009	**Virginia Tanji, MLS** (2011–2012) Librarian University of Hawai’i at Manoa Member, NLM Board of Regents tanji@hawaii.edu
**Robin Featherstone, MLS** (2012) Liaison Librarian (Medicine) Life Sciences Library, McGill University Montréal, Québec H3G 1Y6 robinfeatherstone@mcgill.ca	**Bailus Walker, MD** (2011–2012) Professor, Environmental and Occupational Medicine and Health Policy Howard University College of Medicine bailusw@comcast.net
